# Safety and Efficacy of the Supreme Biodegradable Polymer Sirolimus-Eluting Stent in Patients With Diabetes Mellitus

**DOI:** 10.1016/j.jscai.2022.100033

**Published:** 2022-04-11

**Authors:** Alexander Thomas, Dean J. Kereiakes, Andreas Baumbach, Stephan Windecker, Cody Pietras, Ovidiu Dressler, M. Ozgu Issever, Michael Curtis, Barry Bertolet, James P. Zidar, Pieter C. Smits, Victor Alfonso Jiménez Díaz, Brent McLaurin, Ángel Cequier, Akihiko Takahashi, Louis A. Cannon, Giovanni Amoroso, Tsunekazu Kakuta, Shigeru Saito, Martin B. Leon, Alexandra J. Lansky

**Affiliations:** a Division of Cardiology, Yale School of Medicine, New Haven, Connecticut; b Christ Hospital Heart and Vascular Center, Cincinnati, Ohio; c Centre for Cardiovascular Medicine and Devices, William Harvey Research Institute, Queen Mary University of London and Barts Heart Centre, London, United Kingdom; d Department of Cardiology, Bern University Hospital, Inselspital, University of Bern, Switzerland; e Cardiovascular Research Foundation, New York, New York; f University of Calgary, Alberta, Canada; g Cardiology Associates of North Mississippi, Tupelo, Mississippi; h North Carolina Heart and Vascular, University of North Carolina, Raleigh, North Carolina; i Maasstad Ziekenhuis, Rotterdam, the Netherlands; j Hospital Álvaro Cunqueiro, Vigo, Spain; k AnMed Health Medical Center, Anderson, South Carolina; l Bellvitge Hospital, University of Barcelona, IDIBELL, Spain; m Sakurakai Takahashi Hospital, Kobe, Japan; n Cardiac and Vascular Research Center of Northern Michigan, Petoskey, Michigan; o Onze Lieve Vrouwe Gasthuis, Amsterdam, the Netherlands; p Tsuchiura Kyodo Hospital, Tsuchiura, Japan; q Shonan Kamakura General Hospital, Kamakura, Japan; r College of Physicians and Surgeons, Columbia University, New York, New York

**Keywords:** Diabetes mellitus, drug-eluting stents, coronary revascularization, outcomes

## Abstract

**Background:**

Patients with diabetes mellitus (DM) have worse outcomes following percutaneous coronary intervention than nondiabetic patients. The novel Supreme DES is a biodegradable polymer sirolimus-eluting stent designed to synchronize early drug delivery, limiting the potential for long-term inflammatory response. The purpose of this study was to evaluate the safety and efficacy of the Supreme DES in patients with DM.

**Methods:**

This is a prespecified analysis of the diabetic subgroup from the PIONEER III randomized (2:1), controlled trial, comparing the Supreme DES with a durable polymer everolimus-eluting stent (DP-EES). The primary safety and efficacy composite endpoint was target lesion failure at 1 year, a composite of cardiac death, target vessel myocardial infarction, or clinically driven target lesion revascularization.

**Results:**

The PIONEER III trial randomized 1629 patients, of which 494 (30.3%) had DM with 331 (398 lesions) randomly assigned to Supreme DES and 163 (208 lesions) to DP-EES. Among patients with DM, target lesion failure at 1 year was 6.1% (20/331) with Supreme DES vs 3.7% (6/163) with DP-EES (hazard ratio = 1.65; 95% confidence interval = 0.66-4.10, *P* = .28). The composite of cardiac death or target vessel myocardial infarction was 3.3% (11/331) with Supreme DES and 3.7% (6/163) with DP-EES (hazard ratio = 0.90; 95% confidence interval = 0.33-2.44, *P* = .83). There were no significant differences in other secondary endpoints.

**Conclusions:**

This prespecified substudy of the PIONEER III trial demonstrated the relative safety and efficacy of the novel Supreme DES when compared with commercially available DP-EES in diabetics at 1 year. Longer term follow-up will be required to ensure continued safety and efficacy of the Supreme DES.

## Highlights

• Patients with DM treated with the Supreme DES or DP-EES had similar rates of MACE and probable/definite stent thrombosis at 1 year.
• This prespecified substudy of the prospective, multicenter, randomized trial supports the safety and efficacy of the Supreme DES at 1 year following PCI in patients with DM.
• Longer term follow-up will be required to ensure continued safety and efficacy of the Supreme DES.

## Central Illustration

**Figure:**
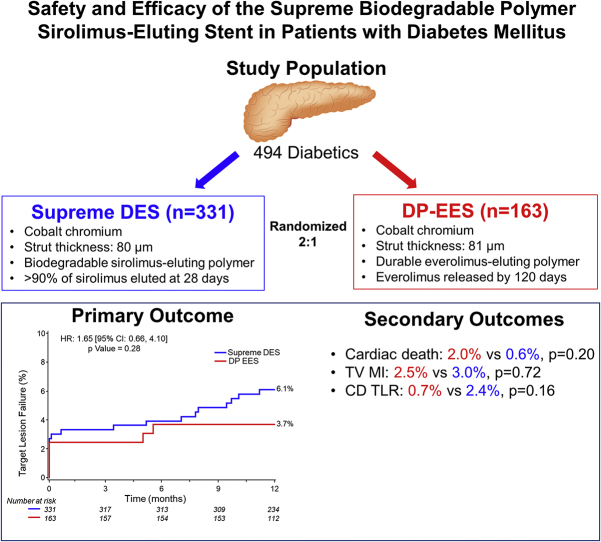

Patients with diabetes mellitus (DM), especially those treated with insulin,^1^^,^^2^ undergoing contemporary percutaneous coronary intervention (PCI) with drug-eluting stents (DES) are at an increased risk for adverse ischemic events including myocardial infarction (MI), stent thrombosis, and restenosis, as well as both cardiac and noncardiac death.^3^, ^4^, ^5^, ^6^ Patients with DM comprise 20% to 30% of the population undergoing PCI,^6^^,^^7^ and as the prevalence of DM continues to rise,^8^ the relative safety and efficacy of novel DES should be evaluated in this high-risk population.

The pathophysiologic mechanisms underlying poor outcomes among diabetic patients following PCI have been extensively studied and, although not completely understood, include differences in atherosclerotic plaque composition, inflammatory response, lesion length and complexity, negative remodeling, and vessel size.^7^^,^^9^, ^10^, ^11^

Technical advances of contemporary DES including thinner struts, more bioinert and compatible polymer coatings, and antiproliferative agents have been designed to minimize arterial injury, decrease inflammation, and suppress smooth muscle proliferation.^12^^,^^13^ Patients with DM however continue to have worse outcomes after DES placement, largely driven by high rates of target lesion revascularization which are almost 2-fold higher at 1 year.^3^^,^^13^^,^^14^ Permanent polymer DES coatings are associated with hypersensitivity, inflammation, neoatherosclerosis, and thrombosis, contributing to restenosis and stent thrombosis.^15^ While antiproliferative drugs inhibit smooth muscle cell proliferation, prolonged drug delivery can delay endothelialization and stent healing and may contribute to stent thrombosis and late DES failure,^11^^,^^16^, ^17^, ^18^ both of which are amplified in the diabetic population.^5^ Biodegradable polymer–coated stents designed to limit the extended inflammatory vascular response have not demonstrated benefits in clinical outcomes in general^19^^,^^20^ or in patients with DM^13^ compared with durable polymer DES. This may be explained in part by the fact that most biodegradable polymer DES have prolonged polymer degradation times ranging from 3 to 18 months, which may delay healing^19^^,^^21^^,^^22^ and lead to persistent long-term stent failure, with target lesion failure (TLF) rates of 10% at 1 year and 30% at 5 years among patients with DM.^13^

The Supreme DES (SINOMED) is a biodegradable polymer DES designed to degrade and deliver sirolimus early within 4 to 6 weeks.^23^ This abbreviated drug delivery sequence allows earlier re-endothelization of the stent with restoration of biologic processes inherent to endothelial tissue that suppress thrombosis and smooth muscle cell proliferation. This prespecified substudy of the PIONEER III trial was designed to evaluate the relative safety and efficacy of the Supreme DES compared with contemporary durable polymer everolimus-eluting stents (DP-EES) among patients with DM.

## Methods

### Design and participants

PIONEER III (NCT03168776) was a prospective, randomized, single-blind, international, multicenter trial conducted at 74 investigational sites across North America, Europe, and Japan. This prespecified subanalysis includes all patients with DM.^21^ DM was defined by the investigators based on the medical history and medical treatment for DM. The full design of the study has been previously described.^21^ Adult men and nonpregnant women aged 20-99 years who presented with symptomatic acute unstable angina, non–ST-segment elevation MI, or chronic ischemic syndromes with evidence of ischemia were included. Notable exclusions were patients presenting with ST-segment elevation MI, unprotected left main coronary artery disease, known left ventricular ejection fraction <30%, or cardiogenic shock. Patients were randomized in a 2:1 ratio, stratified by DM status, to treatment with either Supreme DES or DP-EES. Dual antiplatelet therapy consists of aspirin and a P2Y12 inhibitor for ≥6 months after PCI in chronic coronary syndromes and ≥12 months after PCI for acute coronary syndromes in accordance with published guidelines.^24^^,^^25^ This study was approved by the institutional review board or ethics committee at each site, and informed consent was obtained prior to participation.

### Device description

The Supreme DES is a balloon-expandable, biodegradable polymer, sirolimus-eluting coronary stent system targeting early vascular healing. The biodegradable poly lactic-co-glycolic acid (PLGA) polymer is bonded by a poly n-butyl methacrylate (PBMA) basecoat to the metal surface of the stent by a proprietary electrografting (eG Coating; SINOMED) process. The PLGA polymer has a 50:50 lactide-to-glycolide (L:G) ratio that resorbs in 45-60 days. The eG PBMA coating is designed to minimize polymer flaking or cracking and prevent stent corrosion.^21^ The control stent is a DP-EES (XIENCE, Abbott Vascular; Promus, Boston Scientific Corporation) with established safety and effectiveness in diabetics^26^^,^^27^ and a labeled indication for use in this population.^28^, ^29^, ^30^, ^31^

### Endpoints and definitions

The primary endpoint was TLF at 1 year, defined as the composite of cardiac death, target vessel MI, or clinically driven target lesion revascularization. Secondary endpoints included the individual components of the primary TLF endpoint, the composite of death (cardiac or noncardiac) or nonfatal MI, major adverse cardiac events (composite of death, MI, or target vessel revascularization), target vessel failure, periprocedural MI, probable or definite stent thrombosis, any stent thrombosis, as well as early (≤30 days) and late stent thrombosis.^21^ As with the primary endpoint, all secondary endpoints were evaluated at 1 year.

### Statistical analysis

Categorical variables are reported as counts and percentages and compared between treatment groups using the χ^2^ or Fisher exact test. Continuous variables are presented as mean and standard deviation and compared with the 2-sample *t* test. If the data failed to meet the assumption for normality per the Shapiro–Wilk test, then the comparisons were made using the Wilcoxon rank-sum test. Time-to-event outcomes were calculated using Kaplan–Meier methods and compared between groups using the log-rank test. Cox proportional hazards analysis was used to calculate hazard ratios (HRs) with 95% confidence intervals (CIs) and *P* values. Statistical analyses were performed using SAS, version 9.4 (SAS Institute) by the Cardiovascular Research Foundation.

## Results

### Patient and baseline characteristics

Between October 2017 and July 2019, a total of 1629 patients were enrolled in the PIONEER III trial, of which 494 (33.3%) had DM with 331 (398 lesions) were randomly assigned to Supreme DES and 163 (208 lesions) to DP-EES. We present outcomes for the DM patient subgroup. Clinical follow-up at 12 months was completed in 97.6% (323/331) of Supreme DES and 96.9% (158/163) of DP-EES groups. The mean age was approximately 66 years, 28% were female, and 33.6% were on insulin therapy. Baseline clinical and angiographic characteristics were similar between the groups; Supreme DES patients had less stable angina, more single vessels treated and more complex class C lesions (Table 1). Both groups had high lesion and device success rates of ≥99% and ≥96%, respectively. Dual antiplatelet therapy (DAPT) use for Supreme DES and DP-EES groups was 98.5% and 98.8% immediately after procedure, 95.7% and 95.0% at 6 months, 84.0% and 82.7% at 1 year with mean DAPT durations of 329 days and 323 days, respectively. There were no differences in antilipid or antianginal therapies between groups (Supplemental Table 1).

**Table 1:** Baseline clinical and angiographic characteristics in patients with diabetes.

Parameter	Supreme DES (n = 331)	DP EES (n = 163)	Overall (N = 494)	*P* value
Age, years	66.1 ± 9.7	64.9 ± 9.9	65.7 ± 9.7	.18
Male sex	73.7% (244)	68.1% (111)	71.9% (355)	.19
Type I diabetes	4.5% (15)	3.7% (6)	4.3% (21)	.66
Insulin treatment	33.5% (111)	33.7% (55)	33.6% (166)	.96
Hypertension	88.2% (292)	87.1% (142)	87.9% (434)	.72
Hyperlipidemia	87.3% (289)	91.4% (149)	88.7% (438)	.18
Renal disease	12.4% (41)	13.5% (22)	12.8% (63)	.73
Prior myocardial infarction	19.0% (63)	19.6% (32)	19.2% (95)	.87
Prior PCI	35.0% (116)	36.2% (59)	35.4% (175)	.80
Prior CABG	9.1% (30)	6.7% (11)	8.3% (41)	.38
Prior stroke	7.6% (25)	4.3% (7)	6.5% (32)	.17
Atrial fibrillation	2.1% (7)	1.8% (3)	2.0% (10)	1.00
Peripheral artery disease	6.3% (21)	7.4% (12)	6.7% (33)	.67
Current/former smoker	61.3% (203)	53.4% (87)	58.7% (290)	
Clinical presentation				.03
Stable angina	53.2% (176)	58.3% (95)	54.9% (271)	
Unstable angina	20.8% (69)	17.8% (29)	19.8% (98)	
Silent ischemia	13.0% (43)	5.5% (9)	10.5% (52)	
NSTEMI	13.0% (43)	18.4% (30)	14.8% (73)	
Number of diseased vessels				.30
1	70.4% (233)	62.0% (101)	67.6% (334)	
2	18.7% (62)	25.2% (41)	20.9% (103)	
3	9.4% (31)	11.0% (18)	9.9% (49)	
≥4	1.5% (5)	1.8% (3)	1.6% (8)	
Procedural characteristics				
Number of vessels treated per patient	1.10 ± 0.30	1.17 ± 0.37	1.12 ± 0.33	
Multiple vessels treated	10.3% (34)	16.6% (27)	12.3% (61)	.046
Lesions per patient	1.2 ± 0.4	1.3 ± 0.6	1.2 ± 0.5	
1 Target lesion	81.6% (270)	76.1% (124)	79.8% (394)	
2 Target lesions	16.6% (55)	19.0% (31)	17.4% (86)	
3 Target lesions	1.8% (6)	4.9% (8)	2.8% (14)	
Stents per patient	1.2 ± 0.6	1.3 ± 0.6	1.3 ± 0.6	
Femoral access	23.3% (77)	22.7% (37)	23.1% (114)	.89
Radial access	76.4% (253)	76.7% (125)	76.5% (378)	.95
Hemostasis device use	73.7% (244)	74.2% (121)	73.9% (365)	.90
Target vessel location	n = 398 lesions	n = 208 lesions	N = 606 lesions	
Left anterior descending	44.2% (176)	41.8% (87)	43.4% (263)	.57
Left circumflex	25.9% (103)	27.4% (57)	26.4% (160)	.69
Right	29.6% (118)	30.8% (64)	30.0% (182)	.78
Left main	0.3% (1)	0.0% (0)	0.2% (1)	1.00
FFR performed	8.5% (34)	10.1% (21)	9.1% (55)	.53
IVUS performed	18.1% (72)	16.3% (34)	17.5% (106)	.59
ACC/AHA lesion class				
A	5.8% (23/395)	7.4% (15/204)	6.3% (38/599)	.48
B1	22.8% (90/395)	29.4% (60/204)	25% (150/599)	.09
B2	26.6% (105/395)	27.5% (56/204)	26.9% (161/599)	.83
C	44.8% (177/395)	35.8% (73/204)	41.7% (250/599)	.04
B2/C	71.4% (282/395)	63.2% (129/204)	68.6% (411/599)	.051
Calcification (moderate/severe)	38.2% (151/395)	37.7% (77/204)	38.1% (228/599)	.94
Eccentric	29.6% (117/395)	21.1% (43/204)	26.7% (160/599)	.03
Tortuosity	22.3% (88/395)	26% (53/204)	23.5% (141/599)	.39
Bifurcation	22.3% (88/395)	21.1% (43/204)	21.9% (131/599)	.76

Values are median ± standard deviation, % (n), or % (n/N).

ACC, American College of Cardiology; AHA, American Heart Association; CABG, coronary artery bypass grafting; DES, drug-eluting stent; DP, durable polymer; EES, everolimus-eluting stent; FFR, fractional flow reserve; IVUS, intravascular ultrasound; NSTEMI, non–ST-segment elevation myocardial infarction; PCI, percutaneous coronary intervention.

### Outcomes at 1 year

The primary endpoint of TLF at 1 year was 6.1% (20) with Supreme DES and 3.7% (6) with DP-EES (HR = 1.65; 95% CI = 0.66-4.10, *P* = .28) (Central Illustration). There was no difference in the components of the primary outcome including cardiac death (0.6% vs 2.0%; HR = 0.33; 95% CI = 0.05-1.95, *P* = .20), target vessel MI (3.0% vs 2.5%; HR = 1.23; 95% CI = 0.39-3.92, *P* = .72), and clinically driven target lesion revascularization (2.4% vs 0.7%; HR = 3.94; 95% CI = 0.49-31.48, *P* = .16) between the Supreme DES and DP-EES groups (Table 2, Central Illustration).

**Central Illustration:**
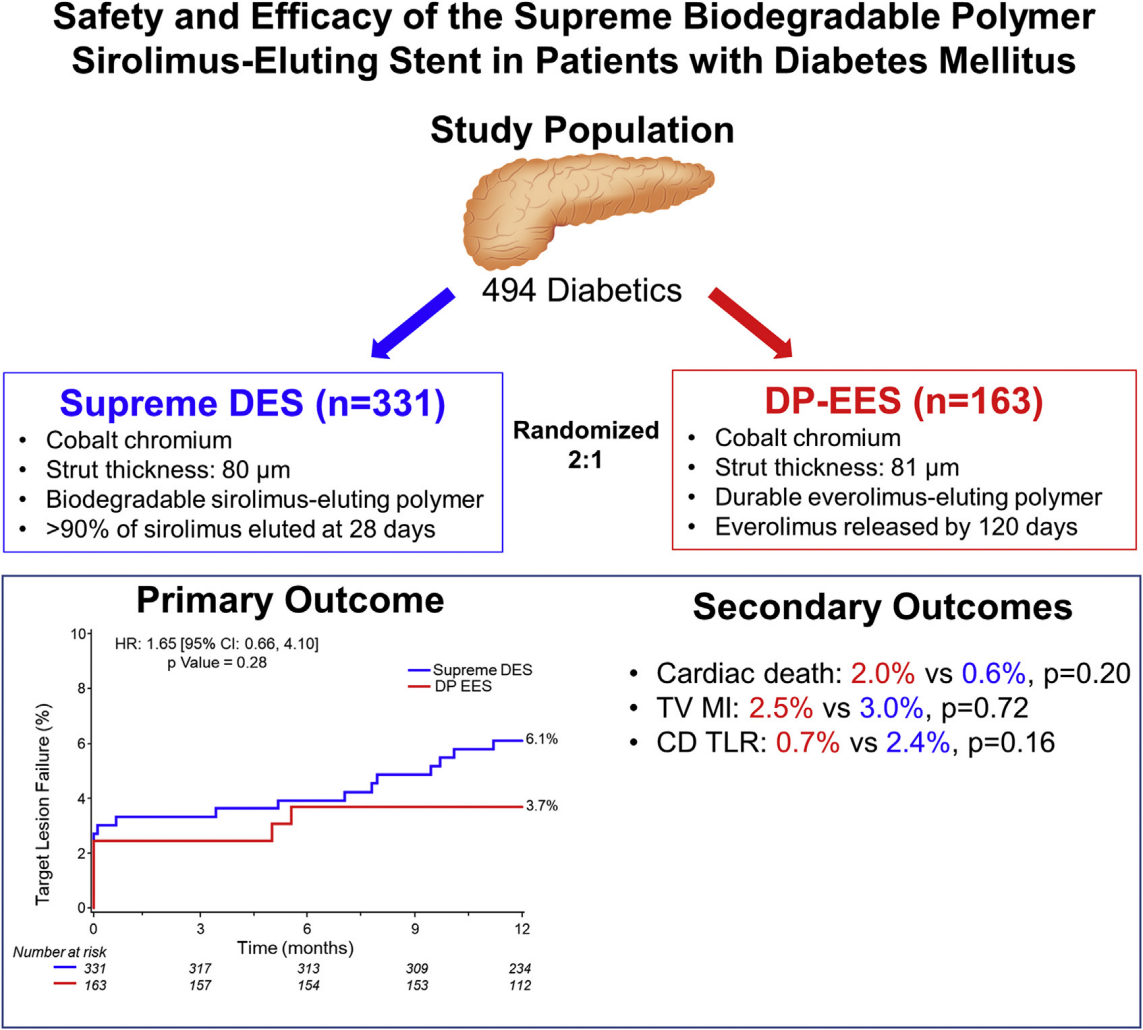
Data for patients who were lost to follow-up or withdrew from the trial before 1 year were censored at the end of follow-up. CD, clinically driven; CI, confidence interval; DES, drug-eluting stent; DP, durable polymer; EES, everolimus-eluting stent; HR, hazard ratio; TLR, target lesion revascularization; TV, target vessel; TVMI, target vessel myocardial infarction.

**Table 2:** One-year clinical outcomes of patients with diabetes.

	Supreme DES (n = 331)	DP EES (n = 163)	Overall (n = 494)	Hazard ratio (95% confidence interval)	*P* value
Target lesion failure (primary outcome)	6.1% (20)	3.7% (6)	5.3% (26)	1.65 (0.66-4.10)	.28
Cardiac death	0.6% (2)	2.0% (3)	1.1% (5)	0.33 (0.05-1.95)	.20
Target vessel myocardial infarction	3.0% (10)	2.5% (4)	2.8% (14)	1.23 (0.39-3.92)	.72
Periprocedural	2.4% (8)	2.5% (4)	2.4% (12)	0.98 (0.30-3.27)	.98
Spontaneous	0.6% (2)	0.8% (1)	0.7% (3)	0.97 (0.09-10.69)	.98
Clinically driven TLR	2.4% (8)	0.7% (1)	1.9% (9)	3.94 (0.49-31.48)	.16
Target vessel failure	6.4% (21)	4.3% (7)	5.7% (28)	1.48 (0.63-3.48)	.36
TLR	2.8% (9)	0.7% (1)	2.1% (10)	4.44 (0.56-35.04)	.12
Major adverse cardiovascular events	7.9% (26)	4.9% (8)	6.9% (34)	1.61 (0.73-3.55)	.23
All death	0.9% (3)	2.6% (4)	1.5% (7)	0.37 (0.08-1.64)	.17
All myocardial infarction	4.9% (16)	3.1% (5)	4.3% (21)	1.57 (0.58-4.30)	.37
Periprocedural	3.0% (10)	2.5% (4)	2.8% (14)	1.23 (0.39-3.93)	.72
Spontaneous	1.9% (6)	1.4% (2)	1.7% (8)	1.46 (0.30-7.25)	.64
All TVR	3.7% (12)	1.4% (2)	2.9% (14)	2.96 (0.66-13.23)	.14
Clinically driven TVR	3.4% (11)	1.4% (2)	2.7% (13)	2.71 (0.60-12.21)	.18
All revascularization	4.9% (16)	3.3% (5)	4.4% (21)	1.58 (0.58-4.31)	.37
Definite/probable stent thrombosis	0.6% (2)	0.0% (0)	0.4% (2)	—	.32
Early (0-30 days)	0.6% (2)	0.0% (0)	0.4% (2)	—	.32
Late (31-365 days)	0.0% (0)	0.0% (0)	0.0% (0)	—	—
Any bleeding (BARC definition)	3.1% (10)	3.1% (5)	3.1% (15)	0.97 (0.33-2.85)	.96
Type 3-5	3.1% (10)	0.6% (1)	2.3% (11)	4.94 (0.63-38.59)	.09

Values are % (n).

BARC, Bleeding Academic Research Consortium; DES, drug-eluting stent; DP, durable polymer; EES, everolimus-eluting stent; TLR, target lesion revascularization; TVR, target vessel revascularization.

**Fig. 1:**
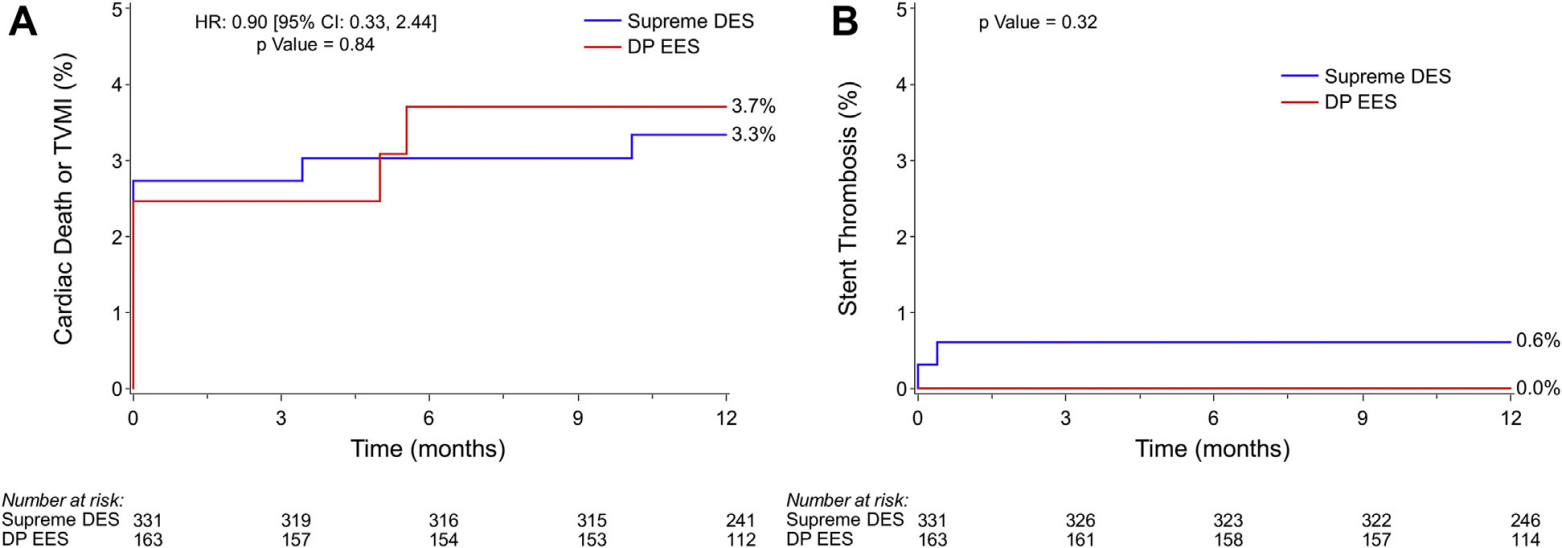
Kaplan–Meier time-to-event curves for secondary outcomes. (**A**) Composite of cardiac death or target vessel myocardial infarction (MI); (**B**) stent thrombosis (definite/probable). Data for patients who were lost to follow-up or withdrew from the trial before 1 year were censored at the end of follow-up. CI, confidence interval; DES, drug-eluting stent; DP, durable polymer; EES, everolimus-eluting stent; HR, hazard ratio; TVMI, target vessel myocardial infarction.

At 1 year, the composite of cardiac death or target vessel MI was 3.3% with Supreme DES and 3.7% with DP-EES (HR = 0.90; 95% CI = 0.33-2.44, *P* = .84) and that of definite/probable stent thrombosis was 0.6% with Supreme DES and 0.0% with DP-EES (*P* = .32) (Fig. 1).

No differences were found between patients treated with Supreme DES or DP-EES for any major secondary endpoints at 12 months including target vessel failure, major adverse cardiac events, all-cause death, any MI, target vessel revascularization, any stent thrombosis, or early stent thrombosis. There was no late definite or probable stent thrombosis in either group (Table 2).

## Discussion

This prespecified substudy of patients with DM from this large-scale, prospective, multicenter, randomized PIONEER III trial supports the safety and efficacy of the Supreme DES. In this randomized DM cohort, rates of TLF, major adverse cardiac events, the composite of cardiac mortality or nonfatal MI, as well as probable or definite stent thrombosis at 1 year were similar between groups. These observations were made in a population of patients with DM, of whom 35% presented with acute coronary syndromes and who had target lesion characteristics similar to those present in recent large-scale studies of diabetics undergoing PCI.^3^^,^^6^

The Supreme DES differs from other biodegradable DES by delivering the antiproliferative drug sirolimus within a short therapeutic window (4-6 weeks) with synchronized polymer matrix degradation allowing early re-endothelization of the stent surface. This accelerated sequence allows for earlier restoration of a functional endothelial barrier with restoration of physiologic vascular functions, which may minimize the inflammatory response.^21^^,^^32^ The benefits derived from a shorter and less pronounced inflammatory response following DES placement are expected to be most apparent between 1 and 5 years. Given these benefits are expected mostly beyond 1 year and that the PIONEER trial was designed to demonstrate noninferiority at 1 year, the current results with Supreme DES demonstrating similar outcomes to DP-EES are reassuring especially in the high-risk DM cohort.

Compared to recently published randomized trials of biodegradable and durable polymer DES among patients with DM, the event rates in the PIONEER trial were lower than previously reported. Specifically, TLF at 1 year among patients with DM with Supreme DES (6.1%) and DP EES (3.7%) was lower than reported for patients with DM in recent trials (range of 6.3%-10.9%).^14^^,^^27^^,^^30^^,^^33^, ^34^, ^35^, ^36^ We also observed lower rates of stent thrombosis (0.6% vs 0.8%-4.6%) and TLR (2.4% vs 3.2%-11.4%) in the Supreme DES group compared with recently published trials.^14^^,^^27^^,^^30^^,^^33^, ^34^, ^35^, ^36^ All other major secondary outcome measures among the Supreme DES group had event rates that fell into the lower third of rates reported in recent biodegradable polymer DES studies among diabetics.^14^^,^^27^^,^^30^^,^^33^, ^34^, ^35^, ^36^ Importantly, the DP-EES control also performed far better than previously reported in diabetic studies.^14^^,^^27^^,^^30^^,^^33^, ^34^, ^35^, ^36^

The numeric difference in TLF between the Supreme DES and DP-EES (6.1% vs 3.7%) was not significant and should be interpreted with caution. The primary difference between groups was driven by rates of TLR, with a TLR rate of 0.7% (1/163) in the DP-EES group, which is notably lower than recently published TLR event rates of 3.2% to 11.4% with DP-EES.^3^, ^4^, ^5^^,^^26^^,^^27^^,^^30^^,^^33^, ^34^, ^35^, ^36^, ^37^, ^38^ Furthermore, the rate of TLR within the diabetic DP-EES population was lower than that within the nondiabetic population from the parent study^21^ and, therefore, likely reflects effects of small sample size compounded by the 2:1 randomization scheme. There were no overt baseline differences seen in our study population compared with recently published studies to explain these findings, nor were there significant differences in rates of medical therapy after PCI including DAPT which had a comparable, if not shorter, average duration than prior studies. It is however important to note that a significant proportion of clinical follow-up was performed between January and July 2020 at the peak of the coronavirus disease 2019 (COVID-19) pandemic. Regardless, the clinical significance of the observed lower event rates remains unclear and longer-term follow-up is necessary to determine its clinical relevance.

## Study limitations

This DM substudy of the PIONEER trial was stratified by DM status and prespecified, therefore preserving randomization within the DM cohort; however, the substudy is limited in its sample size and evaluates only short-term safety and efficacy. Assessment of outcomes at later timepoints will be necessary to establish whether the Supreme DES provides clinical benefit among the diabetic population. This substudy has limited sample size, particularly given the lower-than-expected event rates, and we did not control for multiplicity testing, limiting any definitive conclusions. Patients presenting with ST-segment elevation MI, left main coronary artery disease, and chronic total occlusions were not included in the study population; therefore, results may not be generalizable to these subgroups.

## Conclusion

This prespecified substudy of the prospective, multicenter, randomized PIONEER III trial supports the safety and efficacy of the Supreme DES at 1 year following PCI in patients with DM. Longer term follow-up will be required to ensure the continued relative safety and efficacy of the Supreme DES over time.
