# Supplementary material for: Safety and Efficacy of the Supreme Biodegradable Polymer Sirolimus-Eluting Stent in Patients With Diabetes Mellitus
Source: J Soc Cardiovasc Angiogr Interv. 2022 Apr 11;1(2):100033. doi: 10.1016/j.jscai.2022.100033 (PMC11307809; doi:10.1016/j.jscai.2022.100033)
Supplement: Supplemental Table 1 [file mmc1.docx]

**Supplemental Table 1. Medical Therapy**

| Medication | BuMA DES | DP EES | Overall | P-value^1^ |
| --- | --- | --- | --- | --- |
|  | (N=331) | (N=163) | (N=494) |  |
| **Baseline** |  |  |  |  |
| DAPT | 37.2% (123/331) | 36.2% (59/163) | 36.8% (182/494) | 0.83 |
| Aspirin (monotherapy) | 41.7% (138/331) | 40.5% (66/163) | 41.3% (204/494) | 0.8 |
| P2Y12 inhibitor (monotherapy) | 3.0% (10/331) | 3.7% (6/163) | 3.2% (16/494) | 0.7 |
| Aspirin (any) | 78.9% (261/331) | 76.7% (125/163) | 78.1% (386/494) | 0.58 |
| P2Y12 inhibitor (any) | 40.2% (133/331) | 39.9% (65/163) | 40.1% (198/494) | 0.95 |
| Clopidogrel | 68.4% (91/133) | 61.5% (40/65) | 66.2% (131/198) | 0.34 |
| Ticagrelor | 13.5% (18/133) | 18.5% (12/65) | 15.2% (30/198) | 0.36 |
| Ticlopidine | 0.0% (0/133) | 0.0% (0/65) | 0.0% (0/198) | N/A |
| Prasugrel | 18.0% (24/133) | 20.0% (13/65) | 18.7% (37/198) | 0.74 |
| Beta blocker | 56.8% (188/331) | 60.1% (98/163) | 57.9% (286/494) | 0.48 |
| Statins | 82.5% (273/331) | 81.0% (132/163) | 82.0% (405/494) | 0.68 |
| Nitrates | 32.6% (108/331) | 33.1% (54/163) | 32.8% (162/494) | 0.91 |
| Other lipid lowering agent | 18.7% (62/331) | 21.5% (35/163) | 19.6% (97/494) | 0.47 |
| Warfarin | 0.3% (1/331) | 0.0% (0/163) | 0.2% (1/494) | 1.00* |
| NOAC | 0.0% (0/331) | 0.0% (0/163) | 0.0% (0/494) | — |
| **Post-procedure/discharge** |  |  |  |  |
| DAPT | 98.5% (326/331) | 98.8% (161/163) | 98.6% (487/494) | 1.00 * |
| Aspirin (monotherapy) | 0.6% (2/331) | 0.6% (1/163) | 0.6% (3/494) | 1.00 * |
| P2Y12 inhibitor (monotherapy) | 0.0% (0/330) | 0.6% (1/162) | 0.2% (1/492) | 0.33 * |
| Aspirin (any) | 99.1% (328/331) | 99.4% (162/163) | 99.2% (490/494) | 1.00 * |
| P2Y12 inhibitor (any) | 98.8% (326/330) | 100.0% (162/162) | 99.2% (488/492) | 0.31 * |
| Clopidogrel | 62.3% (203/326) | 54.9% (89/162) | 59.8% (292/488) | 0.12 |
| Ticagrelor | 25.8% (84/326) | 34.6% (56/162) | 28.7% (140/488) | 0.043 |
| Ticlopidine | 0.0% (0/326) | 0.0% (0/162) | 0.0% (0/488) | — |
| Prasugrel | 12.0% (39/326) | 10.5% (17/162) | 11.5% (56/488) | 0.63 |
| Beta blocker | 66.1% (218/330) | 72.4% (118/163) | 68.2% (336/493) | 0.16 |
| Statins | 90.3% (298/330) | 89.6% (146/163) | 90.1% (444/493) | 0.8 |
| Nitrates | 35.8% (118/330) | 30.7% (50/163) | 34.1% (168/493) | 0.26 |
| Other lipid lowering agent | 17.9% (59/330) | 20.2% (33/163) | 18.7% (92/493) | 0.53 |
| Warfarin | 0.3% (1/330) | 0.0% (0/163) | 0.2% (1/493) | 1.00 * |
| NOAC | 0.3% (1/330) | 1.8% (3/163) | 0.8% (4/493) | 0.11 * |
| **6 Months** |  |  |  |  |
| DAPT | 95.7% (311/325) | 95.0% (152/160) | 95.5% (463/485) | 0.73 |
| Aspirin (monotherapy) | 0.9% (3/325) | 3.1% (5/160) | 1.6% (8/485) | 0.12 * |
| P2Y12 inhibitor (monotherapy) | 1.8% (6/325) | 1.9% (3/160) | 1.9% (9/485) | 1.00 * |
| Aspirin (any) | 96.6% (314/325) | 98.1% (157/160) | 97.1% (471/485) | 0.56 * |
| P2Y12 inhibitor (any) | 97.5% (317/325) | 96.9% (155/160) | 97.3% (472/485) | 0.77 * |
| Clopidogrel | 66.2% (210/317) | 58.1% (90/155) | 63.6% (300/472) | 0.08 |
| Ticagrelor | 20.5% (65/317) | 30.3% (47/155) | 23.7% (112/472) | 0.019 |
| Ticlopidine | 0.0% (0/317) | 0.0% (0/155) | 0.0% (0/472) | N/A |
| Prasugrel | 13.2% (42/317) | 11.6% (18/155) | 12.7% (60/472) | 0.62 |
| Beta blocker | 65.6% (212/323) | 71.2% (111/156) | 67.4% (323/479) | 0.23 |
| Statins | 88.9% (287/323) | 89.1% (139/156) | 88.9% (426/479) | 0.94 |
| Nitrates | 31.3% (101/323) | 28.8% (45/156) | 30.5% (146/479) | 0.59 |
| Other lipid lowering agent | 21.4% (69/323) | 24.4% (38/156) | 22.3% (107/479) | 0.46 |
| Warfarin | 0.0% (0/323) | 0.6% (1/156) | 0.2% (1/479) | 0.33 * |
| NOAC | 0.6% (2/323) | 2.6% (4/156) | 1.3% (6/479) | 0.09 * |
| **1 Year** |  |  |  |  |
| DAPT | 84.0% (272/324) | 82.7% (129/156) | 83.5% (401/480) | 0.73 |
| Aspirin (monotherapy) | 10.8% (35/324) | 12.3% (19/155) | 11.3% (54/479) | 0.64 |
| P2Y12 inhibitor (monotherapy) | 2.5% (8/324) | 5.1% (8/156) | 3.3% (16/480) | 0.13 |
| Aspirin (any) | 94.8% (307/324) | 95.5% (148/155) | 95.0% (455/479) | 0.73 |
| P2Y12 inhibitor (any) | 86.4% (280/324) | 87.8% (137/156) | 86.9% (417/480) | 0.67 |
| Clopidogrel | 64.6% (181/280) | 56.9% (78/137) | 62.1% (259/417) | 0.13 |
| Ticagrelor | 21.1% (59/280) | 30.7% (42/137) | 24.2% (101/417) | 0.032 |
| Ticlopidine | 0.0% (0/280) | 0.0% (0/137) | 0.0% (0/417) | N/A |
| Prasugrel | 14.3% (40/280) | 12.4% (17/137) | 13.7% (57/417) | 0.6 |
| Beta blocker | 65.1% (209/321) | 70.3% (109/155) | 66.8% (318/476) | 0.26 |
| Statins | 88.5% (284/321) | 85.2% (132/155) | 87.4% (416/476) | 0.31 |
| Nitrates | 28.7% (92/321) | 30.3% (47/155) | 29.2% (139/476) | 0.71 |
| Other lipid lowering agent | 24.9% (80/321) | 29.0% (45/155) | 26.3% (125/476) | 0.34 |
| Warfarin | 0.3% (1/321) | 0.0% (0/155) | 0.2% (1/476) | 1.00 * |
| NOAC | 1.9% (6/321) | 3.9% (6/155) | 2.5% (12/476) | 0.22 * |
| **Overall DAPT duration** | 329.3 ± (88.3) | 323.9 ± (94.8) | 327.5 ± (90.4) | 0.09 * |

DAPT = dual antiplatelet therapy; NOAC = Novel oral anticoagulant; For categorical variables, the p-value for comparison between two treatment arms is from a chi-squared test. (*) If 20% or more of the expected cell frequencies are less than 5, Fisher’s Exact Test is used to test for difference in proportions
